# Awake Carpometacarpal Denervation for Targeted Pain Relief at the Base of the Thumb Under Local Anesthesia: A Case Report

**DOI:** 10.7759/cureus.94045

**Published:** 2025-10-07

**Authors:** Benjamin F Watzig, Briana Silvestri, Joshua W Hustedt

**Affiliations:** 1 Orthopaedic Surgery, Banner - University Medical Center Phoenix, Phoenix, USA; 2 Sports Medicine, Banner Health, Phoenix, USA; 3 Orthopaedic Surgery, University of Arizona College of Medicine - Phoenix, Phoenix, USA

**Keywords:** basilar thumb arthritis, peripheral denervation, thumb carpometacarpal joint, walant principles, wide awake local anesthesia only

## Abstract

Thumb carpometacarpal (CMC) osteoarthritis is a common source of pain and disability. While denervation has emerged as a motion-preserving alternative to arthroplasty, it is typically performed under regional or general anesthesia. We report the case of a 78-year-old male with systemic scleroderma and pulmonary hypertension who presented with progressive left base of thumb pain. He failed conservative measures, including corticosteroid injection, activity modification, and physical therapy. Examination demonstrated tenderness and pain with the CMC grind. Radiographs revealed advanced degeneration of the CMC and radiocarpal joints, though symptoms were isolated to the CMC joint. After multidisciplinary clearance, he underwent awake CMC joint denervation utilizing local anesthesia in the clinic setting. The procedure was conducted safely without complications. He experienced significant pain relief and functional improvement, without complication, at five-month follow-up.

This case demonstrates the feasibility and safety of performing awake first CMC joint denervation under local anesthetic can provide effective pain relief in appropriately selected patients with advanced CMC arthritis who are poor candidates for general anesthesia.

## Introduction

Thumb carpometacarpal (CMC) osteoarthritis is a common condition, particularly in older adults, and often results in significant pain and functional impairment [1]. Traditional surgical options include trapeziectomy with or without ligament reconstruction and tendon interposition (LRTI), arthrodesis, and implant arthroplasty. As our understanding of hand and wrist neuroanatomy has deepened, so too has our ability to selectively target afferent nerve fibers for therapeutic modulation [2]. Denervation of the CMC joint has emerged as a motion-preserving, lower-morbidity alternative that can provide substantial pain relief and facilitate a quicker return to function [3,4].

Denervation procedures are typically performed in an operating room under regional or general anesthesia. Similar to other procedures about the hand and wrist, performing the procedure under local anesthetic alone has the potential to further reduce costs, anesthetic risk, and recovery time [5,6]. We present a case of primary CMC osteoarthritis treated with awake selective denervation under local anesthetic in an office-based setting.

To our knowledge, there are no prior published reports describing selective CMC joint denervation performed entirely under local anesthesia in an office-based procedure room setting. This case, therefore, represents a novel application of wide-awake local anesthesia principles to thumb CMC denervation. We present this case to illustrate the feasibility and potential benefits of awake selective denervation for thumb CMC osteoarthritis.

## Case presentation

Patient information

A 78-year-old right-hand dominant retired male with a past medical history significant for systemic scleroderma and pulmonary hypertension presented for evaluation of progressive left base of thumb pain over the preceding two years. He was evaluated and treated at Banner - University Medical Center Phoenix in Phoenix, Arizona, USA. The patient denied any preceding trauma to the hand. He reported persistent pain that interfered with daily activities, including gripping, pinching, and household tasks. Conservative management had included activity modification, hand therapy exercises, and corticosteroid injections, all of which provided minimal and transient relief. The patient expressed interest in exploring surgical options while prioritizing a less invasive, motion-preserving approach due to his past medical history.

Examination

On physical examination, the patient demonstrated resolved fingertip ulceration at the pulp of multiple digits, a sequela of systemic scleroderma. There was localized tenderness at the base of the left thumb with pain reproduced on axial load and CMC grind testing. No gross instability was noted. Range of motion at the thumb CMC joint was reduced, particularly in opposition and radial abduction, while distal interphalangeal and metacarpophalangeal (MP) joint motion remained preserved. Neurovascular examination of the hand was normal, and there were no signs of acute inflammatory changes.

Investigations

**Figure 1:**
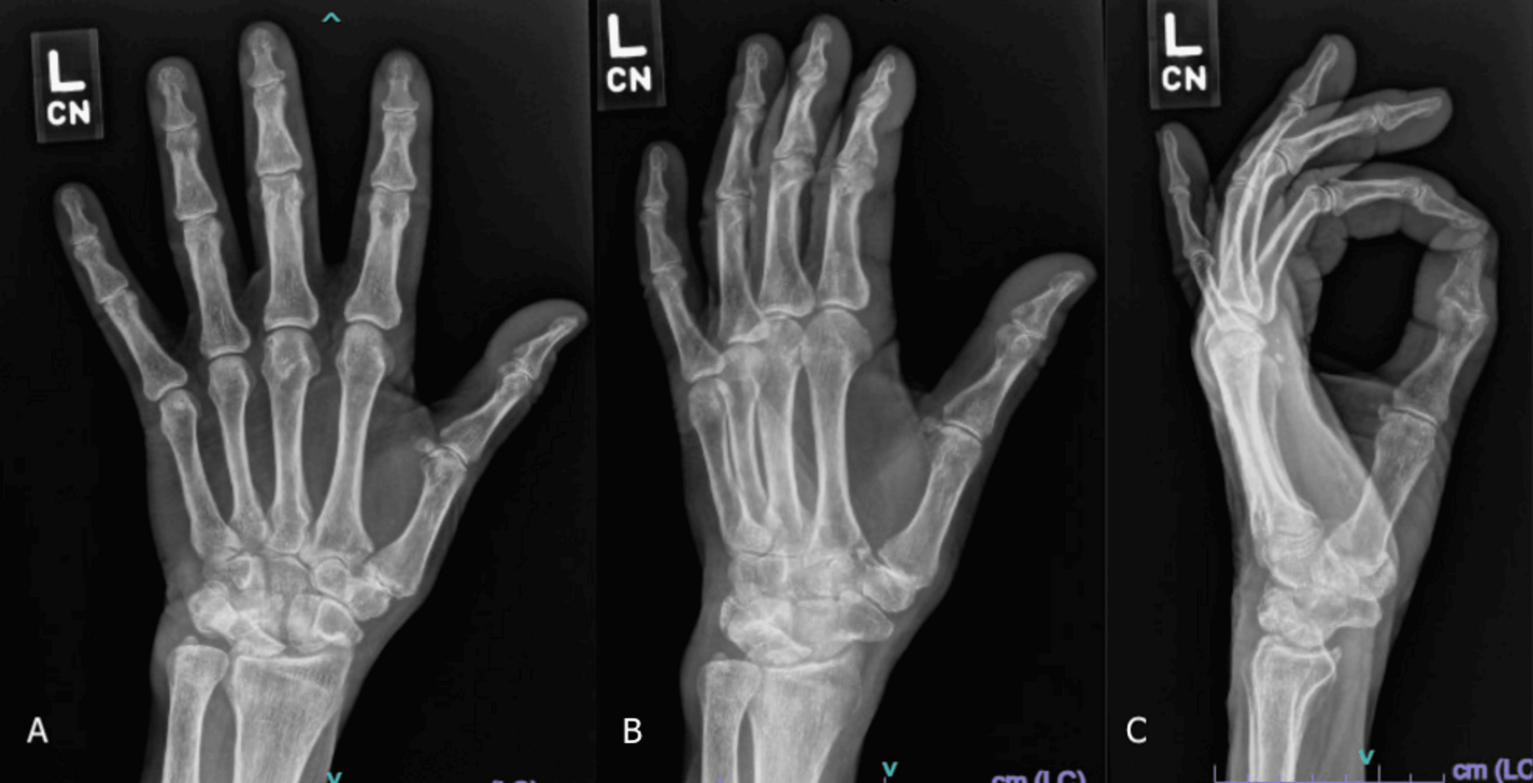
Preoperative left-hand radiographs. (A-C) Posteroanterior (PA), oblique, and lateral views demonstrate significant osteoarthritic changes of the first carpometacarpal (CMC) and radiocarpal joints.

Plain posteroanterior (PA), oblique, and lateral radiographs of the left hand demonstrated advanced degenerative changes at the first CMC joint, characterized by joint space narrowing, subchondral sclerosis, and osteophyte formation. There was also degenerative change noted at the radiocarpal joint, though the patient reported no significant symptoms in this region. Symptoms were localized predominantly to the CMC joint (Figure 1). No advanced imaging, such as MRI or CT, was deemed necessary for surgical planning, as the diagnosis of primary CMC osteoarthritis was clinically and radiographically confirmed.

Preoperative considerations

Given the patient’s medical history, particularly pulmonary hypertension, preoperative clearance was obtained from his pulmonologist prior to any intervention. Due to the increased risk associated with general anesthesia in this patient, a decision was made to perform the procedure entirely under local anesthetic without sedation. The patient was counseled extensively regarding risks, benefits, and alternatives, including more invasive surgical options such as trapeziectomy with LRTI. He expressed a preference for a minimally invasive, motion-preserving approach and agreed to proceed with awake selective CMC denervation in an office-based setting.

Operative technique

**Figure 2:**
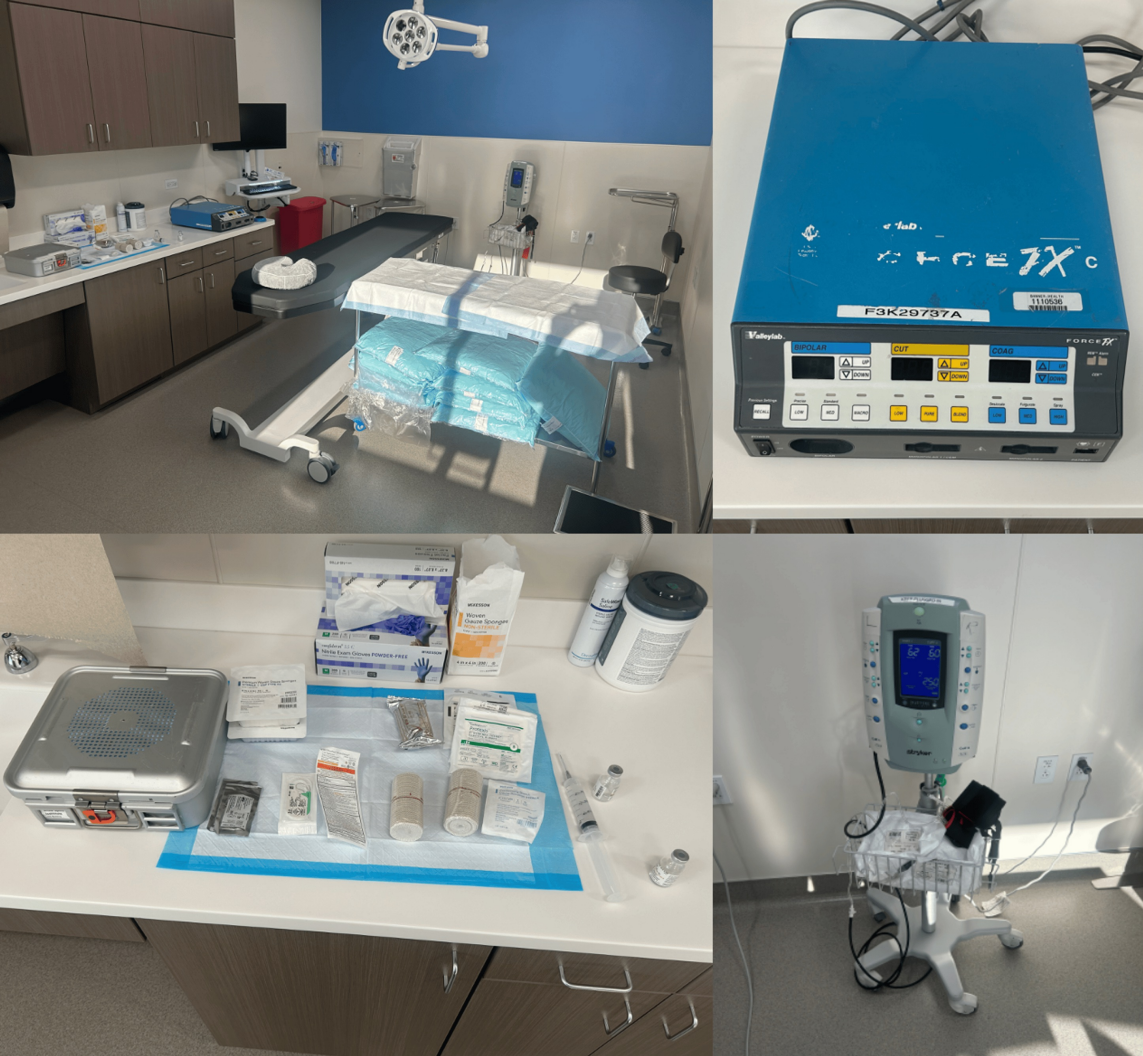
Procedure room setup with supplies.

The procedure was performed in an office-based minor procedure suite using local anesthesia alone, without sedation, consistent with wide-awake local anesthesia principles. However, given the need for close dissection around the radial artery, a tourniquet was applied to provide a bloodless field, thereby enhancing safety and visualization. Our procedure room setup can be seen in Figure 2. After informed consent and marking of the operative site, a pre-operative block was performed with 30cc of 1% lidocaine with epinephrine, with a 30-minute allowance of time before proceeding with surgical intervention (Video 1). A surgical timeout confirmed the correct patient, procedure, and operative site.

**Video 1:** Preoperative local anesthetic administration.

A tourniquet was applied to the upper arm, and the skin over the base of the thumb was prepared in a sterile fashion. The tourniquet was insufflated to 250mmHg.

Dorsal Dissection

A longitudinal incision was made over the first CMC joint. The dorsal sensory branch of the radial nerve was identified, and its articular branch to the CMC joint was isolated and transected.

Thenar Dissection

Subcutaneous dissection proceeded volarly, and the thenar musculature was released along the volar border of the abductor pollicis longus (APL) tendon. The thenar branch of the median nerve was identified, and its articular branch to the CMC joint was carefully dissected, isolated, and transected.

Radial Aspect

Deep dissection exposed the APL and extensor pollicis brevis (EPB) tendon sheaths. The radial artery was identified and protected. The lateral antebrachial cutaneous nerve, running with the radial artery, was identified, and its articular branch to the CMC joint was isolated and transected.

Volar Dissection

Dissection was carried around the volar aspect of the CMC joint and connected with the thenar dissection. The articular branch from the palmar cutaneous branch of the median nerve to the CMC joint was identified, isolated, and transected.

After transection of all articular branches, hemostasis was confirmed, the tourniquet was released with a total time of nine minutes, and the wound was irrigated and closed in layers. The patient tolerated the procedure well, with no intraoperative complications. Soft dressings were applied. A video recording of the procedure demonstrates each step of the dissection and identification of articular nerve branches (Video 2). In addition, a longer and more detailed technique guide with a patient under general anesthesia can be viewed (Video 3).

**Video 2:** Carpometacarpal (CMC) joint denervation performed under local anesthesia.

**Video 3:** Carpometacarpal (CMC) denervation technique. Case presentation and surgical technique for a patient undergoing CMC denervation under general anesthesia.

Postoperative course

The patient reported an immediate reduction in pain at the base of the thumb following the procedure. Early mobilization was encouraged, and he returned to light functional activities within one week. By three weeks postoperatively, he had resumed all activities of daily living without limitations. There were no postoperative complications, including wound infection, edema, hematoma, or sensory deficits.

At the five-month follow-up, the patient remained highly satisfied with the outcome, reporting sustained pain relief localized to the CMC joint. The patient’s Visual Analog Scale (VAS) improved from a preoperative score of 7 to a postoperative score of 2, with a corresponding improvement in the Quick Disabilities of the Arm, Shoulder, and Hand (QuickDASH) score from 63.6 to 29.5. Mild compensatory discomfort was noted at the MP joint, which was managed conservatively and may be addressed with a corticosteroid injection if symptoms persist. Overall, the patient achieved a favorable functional outcome with minimal recovery time and no anesthesia-related complications.

## Discussion

This case illustrates that selective CMC denervation can be performed safely under local anesthesia with a tourniquet in an office-based setting, providing meaningful pain relief while preserving joint motion. Unlike traditional procedures such as trapeziectomy with LRTI, denervation directly interrupts nociceptive input from articular branches without disrupting joint mechanics. This approach can be particularly advantageous in patients with significant comorbidities, such as pulmonary hypertension or systemic sclerosis, for whom general anesthesia or prolonged operative times may carry an elevated risk.

Performing the procedure under local anesthesia with a tourniquet has several potential benefits. General anesthetic risk is minimized, an important consideration in older patients or those with cardiopulmonary conditions [7]. Additionally, eliminating the need for sedation or general anesthesia reduces recovery time and facilitates quicker return to daily activities [8]. Finally, although formal cost analysis was not performed in this case, experience from other hand procedures, including carpal tunnel release and trigger finger release, suggests that office-based local anesthesia procedures can substantially reduce facility and personnel costs compared to the operating room [9]. Overall, in a high-risk patient, we demonstrated a wide-awake, local-only, motion-preserving option for the management of symptomatic CMC osteoarthritis. These findings lay the groundwork for further study and potential broader adoption of office-based denervation techniques for the thumb CMC joint.

## Conclusions

This case demonstrates that selective CMC joint denervation can be safely and effectively performed entirely under local anesthesia in an office-based setting using wide-awake principles. The patient experienced immediate and sustained pain relief, rapid return to functional activities, and no perioperative complications. Compared with traditional surgical approaches performed in the operating room under general or regional anesthesia, this technique may reduce anesthetic risk, shorten recovery time, and lower overall procedural costs, particularly important in medically complex or elderly patients.
